# A large-scale benchmark of gene prioritization methods

**DOI:** 10.1038/srep46598

**Published:** 2017-04-21

**Authors:** Dimitri Guala, Erik L. L. Sonnhammer

**Affiliations:** 1 Stockholm Bioinformatics Center, Department of Biochemistry and Biophysics, Stockholm University, Science for Life Laboratory, Box 1031, 17121 Solna, Sweden

## Abstract

In order to maximize the use of results from high-throughput experimental studies, e.g. GWAS, for identification and diagnostics of new disease-associated genes, it is important to have properly analyzed and benchmarked gene prioritization tools. While prospective benchmarks are underpowered to provide statistically significant results in their attempt to differentiate the performance of gene prioritization tools, a strategy for retrospective benchmarking has been missing, and new tools usually only provide internal validations. The Gene Ontology(GO) contains genes clustered around annotation terms. This intrinsic property of GO can be utilized in construction of robust benchmarks, objective to the problem domain. We demonstrate how this can be achieved for network-based gene prioritization tools, utilizing the FunCoup network. We use cross-validation and a set of appropriate performance measures to compare state-of-the-art gene prioritization algorithms: three based on network diffusion, NetRank and two implementations of Random Walk with Restart, and MaxLink that utilizes network neighborhood. Our benchmark suite provides a systematic and objective way to compare the multitude of available and future gene prioritization tools, enabling researchers to select the best gene prioritization tool for the task at hand, and helping to guide the development of more accurate methods.

High-throughput methods, such as Linkage analysis, Genome Wide Association Studies(GWAS) and RNA interference screens have revolutionized the way we are able to couple genetic variation to diseases. These techniques have become very efficient in connecting large chromosomal regions with genetic disorders, yielding lists of hundreds of potential gene candidates^1^,^2^. However, despite the constant drive for improvement in methodology of these techniques, increasing the experimental efficiency of the methods, the same rate of improvement is not seen when it comes to accuracy^3^ and the rate of False Positives^4^. The sheer volume of candidate genes returned by high-throughput studies makes experimental validation a daunting task. Techniques for prioritizing candidate disease genes are necessary and important in order to maximize the information from these high-throughput experiments, by focusing further experimental efforts on the most promising candidates.

Most gene prioritization tools come equipped with some kind of validation, which is usually just a proof of concept or a case study rather than a real estimation of performance^1^,^5^. The existing benchmarks are highly heterogeneous in terms of set up, performance measures, data sources and test/training gene sets. In an ideal situation, predictions from a prioritization tool should be verified by experimental techniques, assessing the real True/False Positives/Negatives that can be used in calculation of performance. In reality, experimental validations are difficult and retrospective data from the Online Mendelian Inheritance in Man(OMIM)^6^ or GWAS studies is often used. There have been attempts to simulate real world situations by using prospective instead of retrospective knowledge in benchmarking gene prioritization tools^7^. Unfortunately, the prospective validation sets are usually too small to yield statistically significant differences between the tools and only a fraction of these sets have confirmed associations to diseases. Additionally, there are no guarantees that genes assessed as False Positives and True Negatives in these prospective validation attempts one day won’t be discovered to actually belong to the studied disease, thus changing their status to True Positives or False Negatives, respectively, resulting in altered performance assessments.

In order to assess true performance of available gene prioritization algorithms and to select the best method for a particular problem, it is imperative to conduct robust and objective comparisons of available techniques. Currently there is no general strategy for construction of benchmarks, which complicates comparison of tools and their performance evaluation^2^. The diversity of tools, with respect to data sources, makes it difficult to avoid knowledge cross-contamination i.e. selecting benchmark data that has not been used in the design of the tool. This in turn leads to that disease data sets from e.g. OMIM, often used in tool validations and available benchmarks, overestimate the true predictive power of many tools. On the other hand, when prospective data is used instead of retrospective, it is difficult to gather enough data before it gets incorporated into the databases used to develop prioritization tools.

In this paper we propose the use of Gene Ontology(GO)^8^ together with FunCoup^9^,^10^,^11^, as an objective data source for benchmarking gene prioritization tools. Being a database of annotations of gene products, GO has the intrinsic property that gene products annotated with the same term are associated to the same or similar biological processes, cellular components or molecular functions, depending on the ontology. Besides being naturally suited for cross-validation, a model evaluation technique of choice to estimate the generalizability of a tool’s performance, this clustering property improves the robustness of performance measures by decreasing the risk of erroneously assigning a gene product as a False Positive or a True Negative when more knowledge becomes available. In order to further improve the robustness of our benchmark we limited the GO-terms used for cross-validation to a range of certain sizes(number of annotated gene products), similar to earlier attempts^12^. The use of terms, too general or too specific might represent artificial clusters or dilute the natural clustering of annotated genes. In order to provide equal opportunities to the benchmarking tools and to avoid knowledge cross contamination as far as possible, one of the most comprehensive functional association networks, which does not include GO data, FunCoup, was used as the source of interaction data.

## Materials and Methods

### Benchmark

Variations of cross-validation^13^ are some of the most common model evaluation techniques used for assessment of generalizability of classification and prediction algorithms. Cross-validation is a technique where a portion of the data set i.e. the test set, is withheld, while the rest of the data is used for training, or like here, as the input to the algorithm being studied, in an attempt to recover the test set. This process is repeated, varying the test set until all the data has been tested. Cross-validation is usually denoted as X-fold or Leave-X-out cross-validations, where “X” can range from “one” element to some percentage of the data set being held out. In our benchmark, we applied three-fold cross-validation, so the genes annotated with a certain GO-term, were randomly divided into three equally sized parts. Two of the parts were combined and used as a query to the prioritization tool being assessed. The presence and ranking of the held out genes found in the output from the tool i.e. the list of candidate genes, determined the labels, True Positive(TP), False Positive(FP), True Negative(TN), False Negative(FN), respectively, assigned to the genes in the candidate list. Performance measures were calculated for each term and their distributions visualized using Seaborn 0.7.0^14^.

### Performance Measures

The most common way of graphically representing performance of a classifier is by visualizing the rate of TPs(TPR) vs the rate of FPs(FPR) in what is known as a Receiver Operating Characteristic(ROC) curve^15^. Examining the whole ROC curve is not particularly useful since our main interest is focused on the performance of the tools with respect to the top ranked scores. We therefore focused on ROC curves up to the FPR of 10%. The Area Under the ROC curve(AUC), equation(1) where *T* is a threshold parameter,


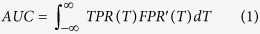


has a convenient probabilistic interpretation as the probability of ranking a randomly chosen positive instance higher than a randomly chosen negative one^15^. It is also a well-established way to capture performance, visualized by a ROC curve, in a single value. In order to make a fair comparison between the tools, focused on the most highly ranked genes, we compared AUCs up to an FPR of 0.02 i.e. partial AUCs(pAUCs), allowing us to study approximately the top 250 candidate genes, which is more than enough for any subsequent experimental validation.

Since gene prioritization tools most often return ranked lists of associated genes it is of interest to determine how high TPs usually rank. However, the length of the candidate list and potential skewness of the distribution of the ranks of TPs prevents us from using the most straightforward way to look at the ranks, i.e. by means of studying the average ranks of TPs. To account for the fact that the ranks of TPs should be skewed towards the top of the list, for any prioritization tool performing better than chance, and to normalize for the length of the candidate list, we calculate the ratio between the median rank of TPs, 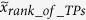 and the total rank, *N*, i.e. Median Rank Ratio(MedRR), equation(2) 7.


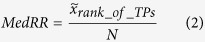


Output from gene prioritization tools is often intended to guide further experiments so it is of importance to assess the ranking capabilities of the said tool. Therefore we also employed a well-established performance measure, used for assessing ranking performance of web searches and other recommender systems, from the field of information retrieval, the Normalized Discounted Cumulative Gain(NDCG), equation(3) 16. NDCG penalizes TPs late in the list to emphasize the importance of retrieving TPs as early as possible.


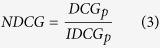


*IDCG*_*p*_ is the ideal *DCGp*, equation(1) and *rel*_*i*_ is the relevance at position *i*.


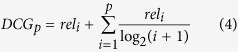


Due to the practical limitations of potential, subsequent experimental analyses it is also useful to assess how well the prioritization tools perform in the top(1% and 10%) of their returned candidate lists.

In our benchmark we used the ROC curves to represent the global performance of a tool i.e. across all GO-terms for one of the ontologies of a specific range. For all the other performance measures we visualize the distribution of values received from calculating performance of each individual GO-term.

The results of our performance measures were not normally distributed, as would be expected for tools aiming to place the most important genes at the top of the candidate list. Therefore we resorted to the Mann-Whitney U test^17^ in evaluating pairwise differences between the tested tools. Correction for multiple hypothesis testing was done using Benjamini-Hochberg procedure^18^.

### Gene Ontology

GO annotations from Biomart Ensembl^19^(Release 69, *H. sapiens*), corresponding to the Ensembl version used in generation of the latest version of FunCoup, were used to extract relationships of GO-terms and annotated genes. GO terms were autocompleted with their parent terms using the acyclic graph of the GO slim ancestry tree(format-version: 1.2, data-version: release/2015-06-03, downloaded on the 31^st^ of May 2015). GO Ontologies: Cellular Component(CC), Molecular Function(MF) and Biological Process(BP), were evaluated separately. GO-terms that are too specific i.e. contain very few genes, may result in candidates classified as False Positives just because the query consists of insufficient number of genes, not enough to capture the complete underlying association pattern. On the other hand, GO-terms annotated with too many genes may be too general, leading to loss of clustering properties offered by the GO-term annotation. It was also of interest to investigate if the level of GO-term specificity had any impact on the results. This resulted in three ranges of GO-terms {10–30}, {31–100} and {101–300} used for evaluation.

### Functional Association Network

FunCoup is one of the most comprehensive networks of functionally associated genes/proteins. It uses redundancy-weighted naïve Bayesian integration to combine different types of evidence of gene/protein coupling, and transfer of orthology information from ten model organisms, to infer functional association between genes/proteins. Combination of complementary data types produces a more complete picture of the interaction landscape^20^ and can mask inherent biases of individual data sources at the same time as cancelling out some of the existing noise. To achieve this, FunCoup uses multiple different types of evidence, including: mRNA co-expression, phylogenetic profile similarity, Protein-Protein Interactions, subcellular co-localization, co-miRNA regulation by shared miRNA targeting, domain interaction, protein co-expression, shared transcription factor binding and genetic interaction profile similarity. There are other comprehensive networks, such as STRING^21^, that could potentially have been used as the underlying network. However, they usually rely on the data from GO which would result in unwanted effects of knowledge cross-contamination in our benchmark, and/or use fewer types of interaction evidence, than FunCoup.

The *Homo sapiens* network from the latest version of FunCoup(v 3.0) was used in the benchmark. In order to reduce noise and avoid spurious links a confidence cutoff of 0.75 was applied to the network, meaning that only links of confidence 0.75 or higher were extracted, resulting in a network of 12 391 genes with 1 123 873 links.

### Gene prioritization tools

MaxLink^22^,^23^ is a network prioritization algorithm that relies on the Guilt-by-association paradigm^24^. It scans a gene/protein network of size, |*V*|, identifies a gene set, *S*, linked to the supplied set of query genes, *Q*, and assigns each candidate gene, *s*∈*S* with a score, corresponding to the number of links to the query set, *ML*. This score is used for ranking the candidate genes. MaxLink utilizes FunCoup to search for genes highly enriched in connections to the query set. In order to avoid highly connected genes from receiving high ranking in the output set, solely based on their high degree, deg(*s*), a connectivity filter, equation(5) is used.


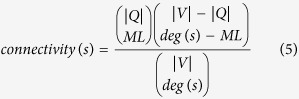


Connectivity filter of 0.5, discarding candidates with *connectivity(s*) ≥ 0.5, was used here.

NetRank^25^ is based on the PageRank algorithm^26^ implemented with priors in the ToppGene suite^27^. The tool represents a Markov chain for a random graph surfer, equation(6). Starting from a set of nodes, 
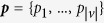
, executing a random walk along the graph with a probability *β*, 0 ≤ *β* ≤ 1 of ending the walk. The probability of starting at a particular node *v* is equal for all the query nodes and zero for all the other nodes i.e. 
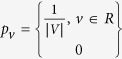
, Where |*V*| is the number of nodes in the network. The probability of ending at a particular node, *v*, is provided by equation(6) 27


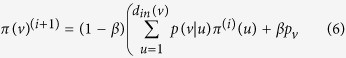


Where *p(v*|*u*) is the probability of transitioning from node *u* to *v* and *d*_*in*_(*V*) is the number of incoming edges. The output is hence all the nodes in the network ranked based on their final probability.

Random Walk with Restart(RWR)^28^, implemented for the purpose of the benchmark and hereby referred to as NetWalk, is the underlying algorithm of many currently available gene prioritization tools^29^,^30^,^31^,^32^,^33^,^34^. It can be visualized in terms of a graph walker starting at a given source node and transitioning to a randomly selected neighbor [Eq.(7)]. There is a probability, *β*(by default set to 0.15), to restart the walk at the source node in every time step. Our implementation uses a vector of initial probabilities, *p*^0^, a vector of current probabilities, ***p***^*t*^ at time step *t*, and the weight matrix, ***W***, defined as, 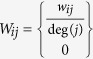 where the original weight of each link, *w*_*ij*_ is normalized with the number of outgoing links, deg(*j*), to advance the time step in equation(7) until ***p***^*t*+1^ ≈ ***p***^*t*^, i.e. differs by no more than 10^−6^.


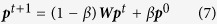


Network propagation algorithm used in the gene prioritization tool PRINCE^34^ and referred to as NetProp is here implemented similarly to RWR but with the weight matrix, ***W***, normalized in a slightly different way, i.e. 
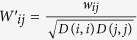
, where *D*_*i,i*_ is the sum of row *i* of *W*_*ij*_.

## Results

In order to compare performance of gene prioritization tools we constructed a benchmark based on functional classes as defined by Gene Ontology(GO) terms. Genes from FunCoup 3.0 were grouped by GO terms for each of the three ontologies(Table 1). The benchmark comprises 5198 GO terms and 12 391 genes.

We applied three-fold cross-validation on each GO-term of a certain size({10–30}, {31–100} and {101–300 genes}) using two thirds of each term’s genes as input to the gene prioritization tool, being assessed, in an attempt to recover the remaining third of the genes. Candidate genes returned by the gene prioritization tool were labeled as True Positives(TPs), False Positives(FPs), True Negatives(TNs) or False Negatives(FNs) based on their membership in the held out set(Alg. 1). Eventually performance for all combinations of GO-term ranges and ontologies were calculated and visualized.
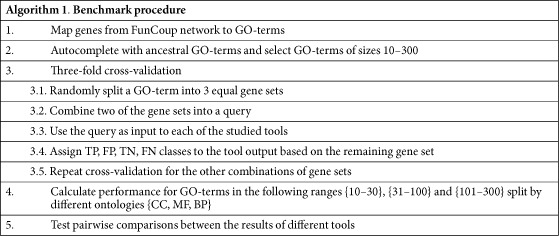


### Median Rank Ratio of True Positives

**Figure 1:**
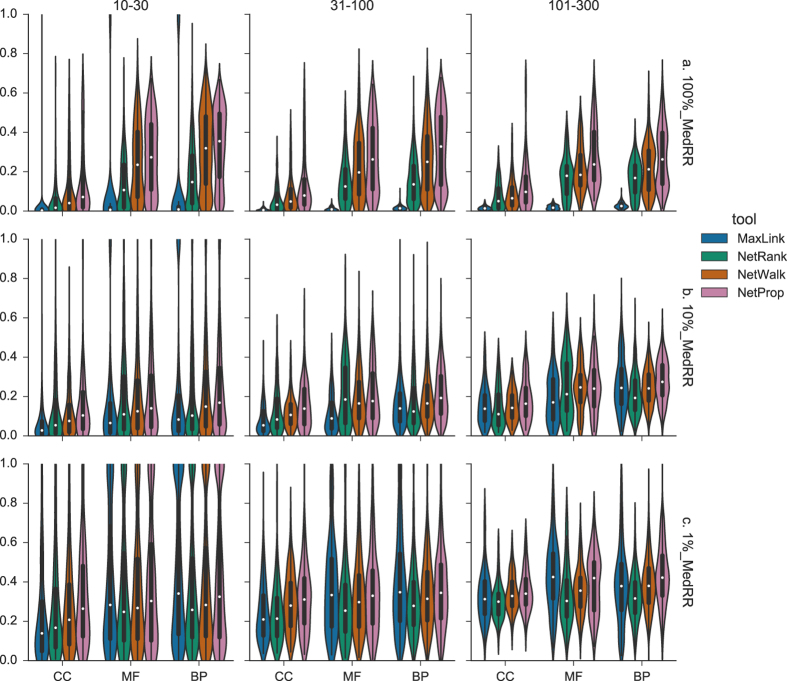
Median Rank Ratio of True Positive(TP) samples(MedRR). MedRR(0 ≤ *MedRR* ≤ 1) for different ranges of GO-terms, i.e. {10–30}, {31–100} and {101–300} was visualized as violin plots, for each tool: MaxLink(blue), NetRank(green), NetWalk(orange) and NetProp(pink), and respective ontology, i.e. Cellular Compartment(CC), Molecular Function(MF), Biological Process(BP). Data points are represented as miniature boxplots within each violin.(**a**) 100% MedRR. Median Rank of TP samples was normalized by the total length of the full candidate gene list.(**b**) 10% MedRR. Median rank of TP samples within the top 10% was normalized by the 10% of the length of the candidate gene list.(**c**) 1% MedRR. Median rank of TP samples within the top 1% was normalized by the 1% of the length of the candidate gene list. Smaller values represent better performance.

The best performance in terms of Median Rank ratio of True Positives(MedRR) for the complete output candidate lists was exhibited by MaxLink with a mean MedRR, 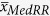, of 0.021 across all ontologies and GO term sizes,(Fig. 1a, Supplemental Table S1). The second best performance was achieved by NetRank with 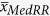 = 0.096, followed by NetWalk 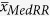 = 0.147, and NetProp 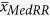 = 0.182. Maxlink was significantly(p < 0.05) better than the other three methods for all of the nine datasets.

Looking at the top 10% of the candidate genes, performance differences become less resolved(Fig. 1b). The only shift in ranking is that Netrank here becomes the best performing method for three of the nine datasets. The difference between MaxLink and NetRank was however not statistically significant for two of these(p = 0.51 and p = 0.08).

For the top 1% of the candidate genes, NetRank performed significantly better than MaxLink in five out of the nine datasets(Fig. 1c). MaxLink was here the best method for CC in the size ranges {10–30} and {31–100}, but not statistically significantly(p = 0.08 and p = 0.90).

In general all tools performed much better than a random prediction(*MedRR* = 0.5). Basing the benchmark on only the top 10% or 1% of the candidate genes decreased MaxLink’s performance drastically, while the other methods were less affected. NetWalk and NetProp even performed the best for the top 10%(Fig. 1b).

### Normalized Discounted Cumulative Gain

**Figure 2:**
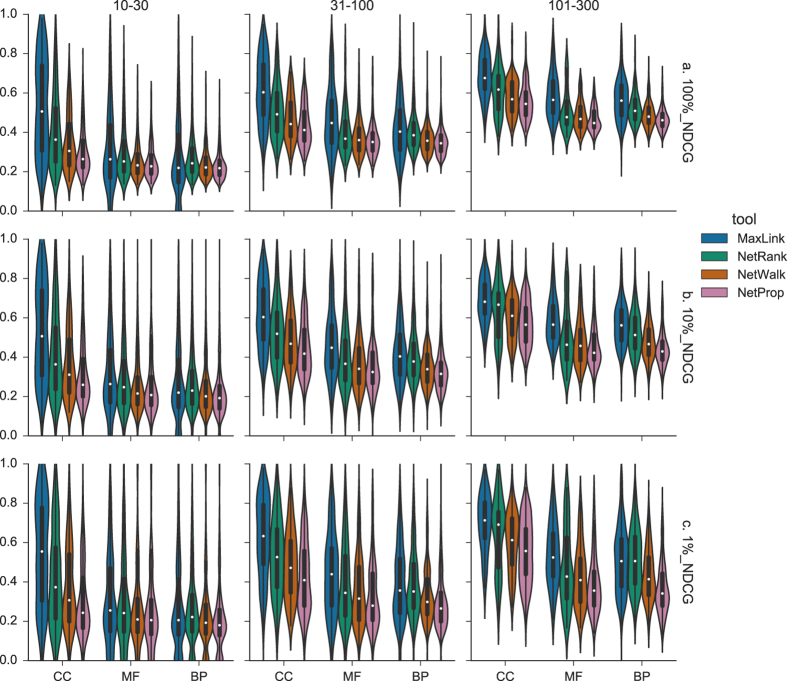
Normalized Discounted Cumulative Gain(NDCG). NDCG looks at the relevance at position *i* in the candidate list and penalizes True Positives(TPs) retrieved late in the list. NDCG ranges between 0 and 1, where 1 is the ideal NDCG thus having the higher values to depict better performance. NDCG is represented as violin plots for different ranges of GO-terms, for each tool: MaxLink(blue), NetRank(green), NetWalk(orange) and NetProp(pink), and respective ontology: Cellular Compartment(CC), Molecular Function(MF), Biological Process(BP). Data points are represented as miniature boxplots within each violin.(**a**) 100% NDCG. NDCG for the full candidate gene list.(**b**) 10% NDCG. NDCG for the top 10% of the candidate gene list.(**c**) 1% NDCG. NDCG for the top 1% of the candidate gene list.

The difference in performance for the chosen GO-term specificity ranges became more apparent for Normalized Discounted Cumulative Gain, or NDCG(Fig. 2a–c). Here performance decreased drastically for the more specific GO-term sets, as the number of genes annotated by a term became smaller. For all datasets except one(BP {10–30}), MaxLink was the best method with mean NDCG, 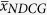 of 0.738, again followed by NetRank at 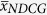 = 0.685, NetWalk at 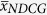 of 0.664 and NetProp at 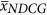 = 0.650, when looking at the complete candidate lists(Fig. 2a, Supplemental Table 1). The difference between MaxLink and NetRank was significant(p < 0.05) for seven of the nine datasets. A minor difference in performance of NetWalk and NetProp was seen also here, again with NetWalk having a slight, but a statistically significant(p < 0.05) advantage for all ontologies but MF where the difference was not statistically significant. As opposed to MedRR, the NDCG performance was almost unchanged when looking at the top 1% or 10% of the candidate genes(Fig. 2b,c), except for a slight widening of the distributions, as expected due to its focus on the relevance of the top candidate genes.

### Receiver Operator Characteristic(ROC) and Area Under the ROC Curves

**Figure 3:**
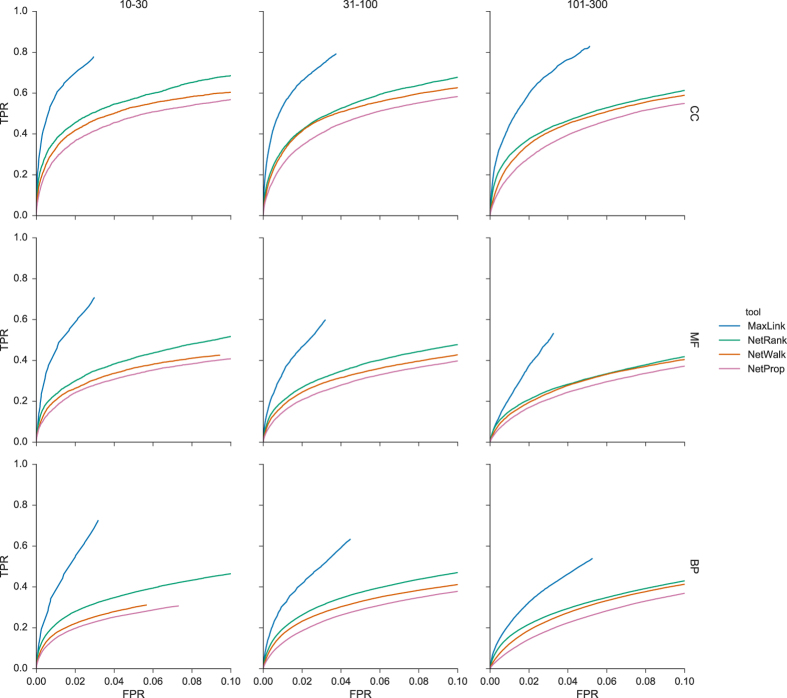
Receiver Operator Characteristic(ROC) curves up to FPR of 0.1 demonstrate the relationship between the True Positive Rate(TPR) and the False Positive Rate(FPR) for each of the hereby evaluated tools: MaxLink(blue), NetRank(green), NetWalk(orange) and NetProp(pink). ROC curves are visualized for all combinations of ontologies i.e. Cellular Compartment(CC), Molecular Function(MF), Biological Process(BP) and GO-term ranges({10–30}, {31–100} and {101–300}).

**Figure 4:**
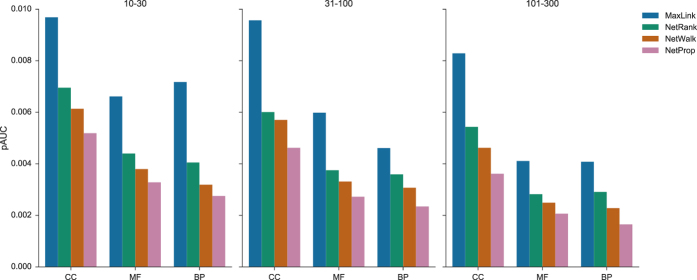
Partial Area Under the ROC curve(pAUC) i.e. the AUC for FPR in the range 0–0.02 for the Cellular Compartment(CC), Molecular Function(MF), and Biological Processes(BP) gene ontologies. Ranges refer to the terms annotating a certain number of genes, e.g. terms annotating 10 to 30 genes, {10–30}. MaxLink(blue), NetRank(green), NetWalk(orange) and NetProp(pink).

The relationship between TPR and FPR for the FPR range of particular interest in gene prioritization, i.e. typically up to an FPR of 0.1, is depicted in ROC curves(Fig. 3). Superior performance for MaxLink across all specificity ranges and ontologies compared to the other tools was apparent both from the ROC curves and the corresponding pAUCs for the FPR up to 0.02(Fig. 4). What also became apparent was that it is possible to get a good resolution of performance even for the very similar NetWalk and NetProp algorithms. NetWalk outperformed NetProp with more or less pronounced difference in all ontology-term range combinations(Figs 3 and 4). NetRank was consistently placed second with less resolved difference towards NetWalk for range {101–300}.

MaxLink did not reach FPR 0.1 in any of the ontology-term range combinations which is why MaxLink curves stop before reaching all the way to the maximum depicted FPR(Fig. 3). This phenomenon is also seen for the other methods but in this FPR range it is only visible for BP of range {10–30}. The reason this happens for MaxLink is that it uses a hypergeometric probability cutoff(FDR = 0.5) to filter out genes assigned as candidates solely based on a high connectivity. This means that all the genes below the cutoff are not reported, and the ROC curve is truncated at this point in the curve. Similar truncations for the other tools usually happen much later, because network based diffusion algorithms such as, NetRank, NetWalk and NetProp, return normalized confidence scores for the whole candidate list. It does however happen when these scores become “indistinguishable” from zero(i.e. rounded off to zero by the Seaborn visualization package).

## Discussion

Although many gene prioritization algorithms have been developed, objective benchmarks for assessing and comparing their performance have been lacking. We have demonstrated the use of the Gene Ontology to construct an unbiased benchmark for network based gene prioritization tools, utilizing FunCoup, one of the most comprehensive functional association networks, as the source of interaction information. The benchmark exploits the fact that gene products with similar functional, compartmental or process characteristics are annotated with the same GO-term. The benchmark contains a broad set of relevant performance measures such as Median Rank ratio of True Positives, Normalized Discounted Cumulative Gain, and Receiver Operator Characteristics with associated Area Under the ROC curve, dealing with ranking capabilities of gene prioritization tools. It studies these performance measures on meaningful ranges of GO terms separated into the different ontologies Cellular Compartment, Molecular Function, and Biological Process.

There are several other performance measures that could have been used in the benchmark i.e. Precision, Recall, Accuracy and correlation measures such as Matthew’s correlation coefficient but we excluded them mostly due to the type of output many of the gene prioritization tools produce. The output is usually a list of all the network genes ranked using a normalized output value for each gene. The excluded performance measures are suitable for measuring performance of classifiers where the numeric value of the output quantifies how strongly the gene belongs to the positive class, and thus serves to determine its class label(TP/FP/FN/TN). However, because the output values are normalized they convey only the relative importance of the genes in the list, making them unsuitable for estimation of performance using the mentioned but excluded performance measures.

An aspect often affecting objectivity of used benchmarks is the fact that benchmark data has topological properties that favor some methods above others. An example of this is that tightly connected networks favor diffusion methods such as NetWalk, NetRank and NetProp over the local neighborhood methods like MaxLink, while when underlying data is sparser, other methods perform better^35^. This could also explain why no single method has yet been shown to dominate universally across different data sources and representations of knowledge^4^. Since the source of functional gene interactions used in this benchmark can be substituted for another suitable gene/protein network, this will allow for an unbiased selection of the best tools for the prioritization task and data at hand. However desirable, this feature is out of scope for this study, but is in the future plan of development of this project. Additionally there are tools that operate on heterogeneous networks utilizing phenotype interactions on top of gene interactions, or interactions with other types of nodes like miRNA^36^,^37^. Our benchmark in its present form is not currently equipped to utilize heterogeneous networks, but this could be a potential extension in the future.

One potential limitation of our approach is that we do not use the whole human genome. This stems from the fact that the FunCoup network with the confidence cutoff of 0.75 only encompasses interactions between 12 391 genes and GO is not comprehensive thus lacking depth of annotation for some genes. Focusing on GO-terms with the number of annotations between 10 and 300 results in 10 346 genes used in our benchmark. When looking at the distribution of annotations across the genes in FunCoup(Supplemental Fig. 1) we see that although it is shifted towards fewer annotations, the majority of genes have between 10 and 150 annotations. When the GO-term cutoff(10–300) is applied the distribution remains similar, with the exception that many genes with fewer than 10 annotations are excluded(Supplemental Fig. 1). Because of the use of GO it is inevitable that genes having more annotations(being potentially more studied) have a bigger impact on our benchmark due to their presence in more training-/test sets. As GO acquires more annotations our benchmark will include more and more genes. In addition, the same genes and annotations were used to train and test the different prioritization tools providing them with equal opportunities for correct prioritizations. The number of genes and annotations used seems also to be sufficient to distinguish between prioritization techniques that are considered very similar, supporting our use of this subset of genes. Finally, GO and FunCoup are among the most comprehensive sources of annotations and protein interactions, respectively, resulting in the most comprehensive gene prioritization benchmark to our knowledge.

Another potential caveat of our benchmark derives from the fact that both GO and FunCoup depict relations between genes, whether through similar annotations, in GO, or through functional association, in FunCoup. Although the data that GO and FunCoup use to achieve this, comes from different sources, acquired and compiled using different frameworks, there may be some overlap in the final outcome. This is something we have tried to assess by depicting the Jaccard normalized GO-term overlap(JNTO) between genes in the FunCoup network as a function of the shortest path(SP) between the genes(Supplemental Fig. 2). Our analysis points to that genes closer in the network appear to have more GO-terms in common(Supplemental Fig. 2). The median JNTO for direct neighbors(SP = 1) is about 50% larger than for next-neighbors(SP = 2). This difference could be attributable to FunCoup trying to connect functionally related genes. The JNTO levels out already at SP = 3, which has the same median as SP = 10. Interestingly, even genes without any path between them share GO-terms to a level which is about half of that seen for direct neighbors, suggesting that the captured relations between genes may have fundamental differences in FunCoup and GO.

To further investigate any potential dependence between GO and FunCoup we looked at the distribution of GO-terms as a function of the degree in FunCoup(Supplemental Fig. 3) where genes from the Human genome not present in FunCoup at this confidence cutoff are assigned a degree of 0. We see that genes not present in FunCoup have slightly fewer GO-term annotations, and the number of annotations increases with the degree. However there are many outliers and the increase could be attributable to pseudogenes lacking both interactions in FunCoup and GO annotations as well as other factors.

A recent gene prioritization study compared a method based on local network neighborhood to a method based on network diffusion, using phenotypes^38^. In this comparison the network diffusion tool was superior for the majority of phenotypes when looking at the overall performance measured by and AUC and illustrated in ROC curves. However when studying the most relevant part of gene prioritization output i.e. the top ranked genes, the local neighborhood based method was superior. This result supports the superior performance of MaxLink we have observed in our benchmark when looking at performance measures focused on the most relevant part of the output from gene prioritization tools.

Diffusion tools studied in this paper have previously been compared to each other^7^, but on a dataset containing prospective data from OMIM. In terms of ranking candidate genes, NetRank was found to perform slightly better than NetWalk tightly followed by NetProp(Table 2). In terms of ROC curves and AUCs(calculated slightly differently than here) NetWalk performed slightly better than the comparable NetProp and NetRank. However the prospective dataset used in this comparison was too limited(42 genes) to draw any definitive conclusions. Besides, as with all comparisons involving OMIM, gene labels(TP/FP/TN/FN) may change when more knowledge is acquired. In our benchmark, performance results support and extend the previous findings, now allowing for statistically significant differentiation between the tools.

The tools examined here were used with their default settings. Optimization of parameters could have yielded potentially different results. However, the fact that we study performance at different ontologies and in different ranges of GO-terms lends credibility to the robustness of the results when they agree across the different settings. Optimization of tool parameters for different benchmark modes is out of scope for this study.

Many more than the four tools, benchmarked here are available for gene prioritization. Some, including, a Conditional Random Field based algorithm^39^, Endeavour^40^ and PINTA^41^ have demonstrated good performance on a subset of metrics tested here^1^,^39^. These and many other methods rely on annotations and or phenotype profiles while our interest lies in being able to assess algorithms which rely solely on the underlying interaction network without the addition of annotations which may bias results towards the more studied genes. Therefore we have limited our tool selection to MaxLink that uses local gene network information and state-of-the-art diffusion tools, NetRank, NetWalk and NetProp. This provides an idea of the level of performance to use as a reference when constructing and testing out new gene prioritization tools.

A limitation of our benchmark stems from the fact that some gene prioritization tools use GO(none use FunCoup to our knowledge), as a source of evidence in order to make predictions. Such tools would gain an unfair advantage in our benchmark due to the circular use of annotations, both in the tool’s algorithm, for prioritization, and in our benchmark when testing. However the gene prioritization tools used here are all network agnostic and do not use any source of annotation to enhance their prediction. Their performance is solely based on the underlying network algorithm and the supplied network. As long as these tools are given the same network and the same training and testing data, a comparison of their performance is assessed without bias. Similar criteria would be preferred for the other tools where this benchmark is to be applied.

Additionally, our benchmark comes equipped with two implementations of the most used state-of-the-art diffusion algorithms, Random Walk with Restart(NetProp and NetWalk) to provide an idea of the high level of performance to use as a reference when constructing and testing out new gene prioritization tool.

By proposing this way of objectively measuring gene prioritization performance, we provide a systematic and objective way to compare the multitude of available and future tools within this problem domain.

## Additional Information

**How to cite this article**: Guala, D. and Sonnhammer, E. L. L. A large-scale benchmark of gene prioritization methods. *Sci. Rep.* **7**, 46598; doi: 10.1038/srep46598(2017).

**Publisher's note:** Springer Nature remains neutral with regard to jurisdictional claims in published maps and institutional affiliations.

## Supplementary Material

Supplementary Information

## Figures and Tables

**Table 1:** Number of terms for each ontology-range pair.

	GO-term ranges		
	{10–30}	{31–100}	{101–300}
Cellular Compartment	233	176	95
Molecular Function	459	248	101
Biological Process	2087	1219	580

Cellular Compartment(CC), Molecular Function(MF), Biological Processes(BP). Ranges refer to the terms annotating a certain number of genes e.g. terms annotating 10 to 30 genes, {10–30}.

**Table 2:** Previous performance comparisons.

	Original paper	Börnigen *et al*.^7^		Current benchmark	
	AUC/%	Ranking	AUC/%	Ranking	AUC/%
NetRank	92	16.8	66	3–18	74–91
NetWalk	98	22.11	71	5–25	66–79
NetProp	91	22.97	67	8–32	63–78

Ranking is normalized to lie in the range of 1 and 100. For the current benchmark a range of MedRR is used in order to account for all combinations of ontologies and GO-term ranges. AUC is in percent.
