# Supplementary material for: A large-scale benchmark of gene prioritization methods
Source: Sci Rep. 2017 Apr 21;7:46598. doi: 10.1038/srep46598 (PMC5399445; doi:10.1038/srep46598)

# A large-scale benchmark of gene prioritization methods

Dimitri Guala* and Erik L.L. Sonnhammer

Stockholm Bioinformatics Center, Department of Biochemistry and Biophysics, Stockholm University, Science for Life Laboratory, Box 1031, 17121 Solna, Sweden

*Corresponding author: Dimitri Guala
Address: Science for Life Laboratory, Box 1031, SE-17121 Solna, Sweden
Email: dimitri.guala@scilifelab.se
Tel: +46-737-440457

## Supplementary information

Supplemental Table S1
Supplemental Figure 1
Supplemental Figure 2
Supplemental Figure 3

**Supplementary Table S1, Pairwise comparisons across all performance measures**

| | | | | | MedRR | | | NDCG | | |
|---|---|---|---|---|---|---|---|---|---|---|
| **Top fraction** | **Term range** | **Ontology** | **tool X** | **tool Y** | **Median X** | **Median diff** | **p-value** | **Median X** | **Median diff** | **p-value** |
| **100%** | **10-30** | **CC** | maxlink | netprop | 0,0028 | -0,0688 | 5,06E-50 | 0,5065 | 0,2420 | 3,54E-24 |
| | | | maxlink | netrank | 0,0028 | -0,0141 | 1,13E-17 | 0,5065 | 0,1432 | 1,05E-07 |
| | | | maxlink | netwalk | 0,0028 | -0,0381 | 1,55E-38 | 0,5065 | 0,2008 | 1,28E-15 |
| | | | netprop | netrank | 0,0715 | 0,0547 | 6,53E-14 | 0,2644 | -0,0988 | 4,97E-09 |
| | | | netprop | netwalk | 0,0715 | 0,0307 | 4,71E-03 | 0,2644 | -0,0412 | 8,03E-04 |
| | | | netrank | netwalk | 0,0168 | -0,0240 | 6,52E-07 | 0,3633 | 0,0576 | 3,13E-03 |
| | | **MF** | maxlink | netprop | 0,0065 | -0,2671 | 5,64E-86 | 0,2634 | 0,0357 | 0,000946 |
| | | | maxlink | netrank | 0,0065 | -0,1005 | 4,39E-49 | 0,2634 | 0,0109 | 0,762848 |
| | | | maxlink | netwalk | 0,0065 | -0,2288 | 2,44E-79 | 0,2634 | 0,0307 | 0,006886 |
| | | | netprop | netrank | 0,2736 | 0,1666 | 1,68E-30 | 0,2277 | -0,0248 | 0,000004 |
| | | | netprop | netwalk | 0,2736 | 0,0383 | 1,02E-02 | 0,2277 | -0,0050 | 0,350493 |
| | | | netrank | netwalk | 0,1071 | -0,1282 | 2,24E-19 | 0,2525 | 0,0198 | 0,000142 |
| | | **BP** | maxlink | netprop | 0,0084 | -0,3470 | 0,00E+00 | 0,2196 | 0,0013 | 6,31E-01 |
| | | | maxlink | netrank | 0,0084 | -0,1392 | 8,75E-198 | 0,2196 | -0,0235 | 4,91E-13 |
| | | | maxlink | netwalk | 0,0084 | -0,3112 | 7,577690e-310 | 0,2196 | -0,0023 | 3,02E-01 |
| | | | netprop | netrank | 0,3553 | 0,2078 | 2,84E-144 | 0,2183 | -0,0248 | 1,26E-36 |
| | | | netprop | netwalk | 0,3553 | 0,0357 | 1,16E-03 | 0,2183 | -0,0036 | 3,47E-03 |
| | | | netrank | netwalk | 0,1476 | -0,1720 | 4,96E-107 | 0,2431 | 0,0211 | 7,84E-23 |
| | **31-100** | **CC** | maxlink | netprop | 0,0054 | -0,0739 | 7,41E-39 | 0,6038 | 0,1918 | 5,59E-23 |
| | | | maxlink | netrank | 0,0054 | -0,0271 | 1,64E-19 | 0,6038 | 0,1118 | 5,29E-08 |
| | | | maxlink | netwalk | 0,0054 | -0,0434 | 5,57E-31 | 0,6038 | 0,1616 | 4,59E-17 |
| | | | netprop | netrank | 0,0793 | 0,0469 | 7,96E-07 | 0,4121 | -0,0800 | 1,04E-07 |
| | | | netprop | netwalk | 0,0793 | 0,0305 | 1,50E-03 | 0,4121 | -0,0302 | 8,67E-03 |
| | | | netrank | netwalk | 0,0324 | -0,0164 | 2,98E-02 | 0,4920 | 0,0498 | 2,50E-03 |
| | | **MF** | maxlink | netprop | 0,0089 | -0,2539 | 6,02E-66 | 0,4477 | 0,0965 | 1,22E-13 |
| | | | maxlink | netrank | 0,0089 | -0,1165 | 1,62E-51 | 0,4477 | 0,0797 | 7,58E-05 |
| | | | maxlink | netwalk | 0,0089 | -0,1877 | 5,31E-61 | 0,4477 | 0,0869 | 3,97E-10 |
| | | | netprop | netrank | 0,2627 | 0,1374 | 3,38E-13 | 0,3512 | -0,0168 | 1,51E-04 |
| | | | netprop | netwalk | 0,2627 | 0,0662 | 1,34E-03 | 0,3512 | -0,0096 | 1,02E-01 |
| | | | netrank | netwalk | 0,1253 | -0,0712 | 3,06E-06 | 0,3680 | 0,0072 | 1,81E-02 |

| | | | | | | | | | | |
|---|---|---|---|---|---|---|---|---|---|---|
| | | | maxlink | netprop | 0,0143 | -0,3139 | 0,00E+00 | 0,4039 | 0,0591 | 1,37E-35 |
| | | | maxlink | netrank | 0,0143 | -0,1216 | 3,52E-266 | 0,4039 | 0,0183 | 1,29E-01 |
| | | BP | maxlink | netwalk | 0,0143 | -0,2360 | 0,00E+00 | 0,4039 | 0,0463 | 4,22E-21 |
| | | | netprop | netrank | 0,3281 | 0,1922 | 2,02E-84 | 0,3448 | -0,0409 | 1,19E-51 |
| | | | netprop | netwalk | 0,3281 | 0,0778 | 1,13E-14 | 0,3448 | -0,0128 | 3,32E-07 |
| | | | netrank | netwalk | 0,1359 | -0,1144 | 2,24E-43 | 0,3856 | 0,0281 | 5,11E-26 |
| | | | maxlink | netprop | 0,0138 | -0,0837 | 6,29E-17 | 0,6763 | 0,1315 | 8,13E-15 |
| | | | maxlink | netrank | 0,0138 | -0,0369 | 2,41E-07 | 0,6763 | 0,0587 | 5,43E-04 |
| | | CC | maxlink | netwalk | 0,0138 | -0,0515 | 1,23E-13 | 0,6763 | 0,1078 | 1,10E-10 |
| | | | netprop | netrank | 0,0975 | 0,0468 | 1,17E-02 | 0,5448 | -0,0727 | 5,30E-05 |
| | | | netprop | netwalk | 0,0975 | 0,0322 | 2,68E-02 | 0,5448 | -0,0236 | 3,99E-02 |
| | | | netrank | netwalk | 0,0507 | -0,0146 | 4,57E-01 | 0,6176 | 0,0491 | 7,47E-03 |
| | | | maxlink | netprop | 0,0170 | -0,2203 | 1,46E-32 | 0,5653 | 0,1179 | 1,54E-14 |
| | | | maxlink | netrank | 0,0170 | -0,1622 | 5,78E-24 | 0,5653 | 0,0877 | 9,16E-07 |
| | 101-300 | MF | maxlink | netwalk | 0,0170 | -0,1671 | 1,55E-31 | 0,5653 | 0,0973 | 1,06E-10 |
| | | | netprop | netrank | 0,2373 | 0,0580 | 5,70E-06 | 0,4474 | -0,0301 | 1,32E-02 |
| | | | netprop | netwalk | 0,2373 | 0,0532 | 1,12E-02 | 0,4474 | -0,0206 | 1,31E-01 |
| | | | netrank | netwalk | 0,1792 | -0,0048 | 1,82E-02 | 0,4776 | 0,0096 | 2,11E-01 |
| | | | maxlink | netprop | 0,0256 | -0,2373 | 2,55E-159 | 0,5614 | 0,0995 | 1,03E-62 |
| | | | maxlink | netrank | 0,0256 | -0,1427 | 4,73E-126 | 0,5614 | 0,0521 | 4,82E-12 |
| | | BP | maxlink | netwalk | 0,0256 | -0,1867 | 8,30E-145 | 0,5614 | 0,0811 | 7,65E-39 |
| | | | netprop | netrank | 0,2629 | 0,0945 | 1,45E-26 | 0,4619 | -0,0474 | 2,25E-39 |
| | | | netprop | netwalk | 0,2629 | 0,0505 | 4,26E-10 | 0,4619 | -0,0184 | 5,01E-10 |
| | | | netrank | netwalk | 0,1684 | -0,0440 | 2,39E-09 | 0,5093 | 0,0291 | 3,60E-14 |
| 10% | | | maxlink | netprop | 0,0276 | -0,0764 | 4,14E-15 | 0,5065 | 0,2470 | 3,59E-22 |
| | | | maxlink | netrank | 0,0276 | -0,0266 | 9,47E-06 | 0,5065 | 0,1428 | 9,41E-07 |
| | | CC | maxlink | netwalk | 0,0276 | -0,0482 | 6,89E-09 | 0,5065 | 0,1957 | 3,31E-13 |
| | | | netprop | netrank | 0,1040 | 0,0498 | 4,86E-03 | 0,2594 | -0,1043 | 2,97E-07 |
| | | | netprop | netwalk | 0,1040 | 0,0281 | 1,79E-02 | 0,2594 | -0,0513 | 1,67E-03 |
| | | | netrank | netwalk | 0,0541 | -0,0217 | 4,20E-01 | 0,3637 | 0,0529 | 1,62E-02 |
| | | | maxlink | netprop | 0,0654 | -0,0747 | 2,37E-09 | 0,2634 | 0,0559 | 5,48E-07 |
| | | | maxlink | netrank | 0,0654 | -0,0442 | 1,08E-06 | 0,2634 | 0,0160 | 1,38E-01 |
| | 10-30 | MF | maxlink | netwalk | 0,0654 | -0,0597 | 3,87E-08 | 0,2634 | 0,0480 | 1,25E-05 |

| | | | | | | | | | |
|---|---|---|---|---|---|---|---|---|---|
| 10-30 | MF | netprop | netrank | 0,1402 | 0,0306 | 5,38E-01 | 0,2075 | -0,0399 | 1,79E-04 |
| | | netprop | netwalk | 0,1402 | 0,0151 | 5,38E-01 | 0,2075 | -0,0079 | 2,40E-01 |
| | | netrank | netwalk | 0,1096 | -0,0155 | 8,52E-01 | 0,2474 | 0,0320 | 4,40E-03 |
| | BP | maxlink | netprop | 0,0829 | -0,0856 | 7,75E-28 | 0,2196 | 0,0277 | 5,10E-16 |
| | | maxlink | netrank | 0,0829 | -0,0201 | 1,92E-06 | 0,2196 | -0,0104 | 3,85E-02 |
| | | maxlink | netwalk | 0,0829 | -0,0660 | 6,29E-20 | 0,2196 | 0,0185 | 3,30E-07 |
| | | netprop | netrank | 0,1685 | 0,0654 | 8,47E-09 | 0,1919 | -0,0381 | 6,08E-33 |
| | | netprop | netwalk | 0,1685 | 0,0196 | 3,61E-02 | 0,1919 | -0,0092 | 1,27E-04 |
| | | netrank | netwalk | 0,1031 | -0,0458 | 8,71E-05 | 0,2300 | 0,0289 | 4,55E-17 |
| 31-100 | CC | maxlink | netprop | 0,0537 | -0,0848 | 3,51E-08 | 0,6038 | 0,1861 | 2,61E-18 |
| | | maxlink | netrank | 0,0537 | -0,0296 | 2,23E-03 | 0,6038 | 0,0850 | 6,26E-06 |
| | | maxlink | netwalk | 0,0537 | -0,0527 | 1,16E-05 | 0,6038 | 0,1359 | 8,29E-13 |
| | | netprop | netrank | 0,1385 | 0,0552 | 2,28E-02 | 0,4178 | -0,1010 | 3,22E-06 |
| | | netprop | netwalk | 0,1385 | 0,0321 | 2,28E-02 | 0,4178 | -0,0502 | 1,90E-02 |
| | | netrank | netwalk | 0,0833 | -0,0231 | 4,52E-01 | 0,5188 | 0,0508 | 9,00E-03 |
| | MF | maxlink | netprop | 0,0886 | -0,0883 | 3,07E-12 | 0,4477 | 0,1232 | 1,19E-15 |
| | | maxlink | netrank | 0,0886 | -0,0964 | 6,28E-11 | 0,4477 | 0,0814 | 1,10E-05 |
| | | maxlink | netwalk | 0,0886 | -0,0763 | 6,28E-11 | 0,4477 | 0,1069 | 3,31E-11 |
| | | netprop | netrank | 0,1769 | -0,0081 | 9,15E-01 | 0,3245 | -0,0419 | 1,12E-03 |
| | | netprop | netwalk | 0,1769 | 0,0120 | 3,70E-01 | 0,3245 | -0,0163 | 1,54E-01 |
| | | netrank | netwalk | 0,1850 | 0,0201 | 3,70E-01 | 0,3664 | 0,0255 | 4,96E-02 |
| | BP | maxlink | netprop | 0,1394 | -0,0531 | 7,59E-21 | 0,4039 | 0,0890 | 1,10E-62 |
| | | maxlink | netrank | 0,1394 | 0,0153 | 7,87E-01 | 0,4039 | 0,0260 | 4,44E-03 |
| | | maxlink | netwalk | 0,1394 | -0,0257 | 3,16E-07 | 0,4039 | 0,0651 | 4,33E-34 |
| | | netprop | netrank | 0,1925 | 0,0685 | 2,79E-17 | 0,3149 | -0,0630 | 1,06E-52 |
| | | netprop | netwalk | 0,1925 | 0,0274 | 4,59E-06 | 0,3149 | -0,0239 | 3,51E-09 |
| | | netrank | netwalk | 0,1241 | -0,0410 | 5,70E-07 | 0,3779 | 0,0391 | 6,47E-24 |
| | CC | maxlink | netprop | 0,1379 | -0,0256 | 0,544183 | 0,6817 | 0,1166 | 1,94E-10 |
| | | maxlink | netrank | 0,1379 | 0,0275 | 0,508077 | 0,6817 | 0,0150 | 5,38E-02 |
| | | maxlink | netwalk | 0,1379 | -0,0041 | 0,657303 | 0,6817 | 0,0717 | 1,41E-06 |
| | | netprop | netrank | 0,1635 | 0,0531 | 0,290362 | 0,5651 | -0,1016 | 4,44E-04 |
| | | netprop | netwalk | 0,1635 | 0,0215 | 0,290362 | 0,5651 | -0,0449 | 7,40E-02 |
| | | netrank | netwalk | 0,1104 | -0,0315 | 0,5982 | 0,6667 | 0,0567 | 2,53E-02 |

| | | | | | | | | | | |
|---|---|---|---|---|---|---|---|---|---|---|
| | | | maxlink | netprop | 0,1703 | -0,0697 | 0,017509 | 0,5653 | 0,1432 | 3,66E-14 |
| | | | maxlink | netrank | 0,1703 | -0,0418 | 0,017509 | 0,5653 | 0,1021 | 7,58E-07 |
| | | | maxlink | netwalk | 0,1703 | -0,0761 | 0,017509 | 0,5653 | 0,1078 | 9,22E-10 |
| | **101-300** | **MF** | netprop | netrank | 0,2400 | 0,0279 | 0,930941 | 0,4220 | -0,0411 | 6,98E-02 |
| | | | netprop | netwalk | 0,2400 | -0,0064 | 0,930941 | 0,4220 | -0,0354 | 2,26E-01 |
| | | | netrank | netwalk | 0,2120 | -0,0344 | 0,930941 | 0,4632 | 0,0057 | 5,31E-01 |
| | | | maxlink | netprop | 0,2436 | -0,0308 | 1,47E-03 | 0,5618 | 0,1327 | 2,50E-79 |
| | | | maxlink | netrank | 0,2436 | 0,0508 | 3,87E-08 | 0,5618 | 0,0491 | 3,02E-09 |
| | | **BP** | maxlink | netwalk | 0,2436 | 0,0023 | 3,12E-01 | 0,5618 | 0,0955 | 2,41E-42 |
| | | | netprop | netrank | 0,2744 | 0,0816 | 1,69E-22 | 0,4292 | -0,0835 | 1,07E-41 |
| | | | netprop | netwalk | 0,2744 | 0,0331 | 3,89E-08 | 0,4292 | -0,0371 | 1,22E-12 |
| | | | netrank | netwalk | 0,1928 | -0,0485 | 2,51E-08 | 0,5127 | 0,0464 | 4,37E-14 |
| **1%** | | | maxlink | netprop | 0,1388 | -0,1253 | 0,000004 | 0,5548 | 0,3116 | 2,66E-21 |
| | | | maxlink | netrank | 0,1388 | -0,0283 | 0,080689 | 0,5548 | 0,1816 | 1,34E-06 |
| | | **CC** | maxlink | netwalk | 0,1388 | -0,0687 | 0,013976 | 0,5548 | 0,2477 | 1,10E-11 |
| | | | netprop | netrank | 0,2642 | 0,0970 | 0,002002 | 0,2431 | -0,1301 | 1,34E-06 |
| | | | netprop | netwalk | 0,2642 | 0,0566 | 0,013976 | 0,2431 | -0,0640 | 2,90E-04 |
| | | | netrank | netwalk | 0,1671 | -0,0404 | 0,387084 | 0,3732 | 0,0661 | 6,75E-02 |
| | | | maxlink | netprop | 0,2830 | -0,0202 | 0,76759 | 0,2553 | 0,0495 | 0,000062 |
| | | | maxlink | netrank | 0,2830 | 0,0350 | 0,452128 | 0,2553 | 0,0133 | 0,277424 |
| | **10-30** | **MF** | maxlink | netwalk | 0,2830 | 0,0155 | 0,452128 | 0,2553 | 0,0459 | 0,001521 |
| | | | netprop | netrank | 0,3032 | 0,0553 | 0,475388 | 0,2057 | -0,0363 | 0,001521 |
| | | | netprop | netwalk | 0,3032 | 0,0357 | 0,475388 | 0,2057 | -0,0036 | 0,214961 |
| | | | netrank | netwalk | 0,2480 | -0,0195 | 0,977435 | 0,2420 | 0,0326 | 0,033774 |
| | | | maxlink | netprop | 0,3410 | 0,0162 | 6,99E-02 | 0,2058 | 0,0259 | 2,18E-18 |
| | | | maxlink | netrank | 0,3410 | 0,0829 | 1,92E-10 | 0,2058 | -0,0157 | 4,69E-02 |
| | | **BP** | maxlink | netwalk | 0,3410 | 0,0580 | 1,01E-06 | 0,2058 | 0,0133 | 4,11E-06 |
| | | | netprop | netrank | 0,3248 | 0,0667 | 1,79E-06 | 0,1799 | -0,0417 | 8,40E-31 |
| | | | netprop | netwalk | 0,3248 | 0,0418 | 1,37E-03 | 0,1799 | -0,0126 | 5,13E-06 |
| | | | netrank | netwalk | 0,2581 | -0,0249 | 8,93E-02 | 0,2216 | 0,0290 | 2,59E-13 |
| | | | maxlink | netprop | 0,2095 | -0,1014 | 0,002508 | 0,6330 | 0,2239 | 3,05E-17 |
| | | | maxlink | netrank | 0,2095 | -0,0041 | 0,90327 | 0,6330 | 0,1067 | 9,96E-05 |
| | | **CC** | maxlink | netwalk | 0,2095 | -0,0703 | 0,003822 | 0,6330 | 0,1614 | 4,08E-11 |

| | | | | | | | | | | |
|---|---|---|---|---|---|---|---|---|---|---|
| | **31-100** | **CC** | netprop | netrank | 0,3108 | 0,0973 | 0,001919 | 0,4091 | -0,1172 | 3,15E-07 |
| | | | netprop | netwalk | 0,3108 | 0,0311 | 0,57558 | 0,4091 | -0,0625 | 5,07E-03 |
| | | | netrank | netwalk | 0,2135 | -0,0662 | 0,003366 | 0,5263 | 0,0547 | 5,76E-03 |
| | | **MF** | maxlink | netprop | 0,3333 | 0,0036 | 0,234603 | 0,4392 | 0,1599 | 5,26E-10 |
| | | | maxlink | netrank | 0,3333 | 0,0793 | 0,000406 | 0,4392 | 0,0948 | 8,61E-03 |
| | | | maxlink | netwalk | 0,3333 | 0,0360 | 0,042707 | 0,4392 | 0,1238 | 1,39E-06 |
| | | | netprop | netrank | 0,3297 | 0,0757 | 0,004742 | 0,2793 | -0,0651 | 4,83E-04 |
| | | | netprop | netwalk | 0,3297 | 0,0324 | 0,227924 | 0,2793 | -0,0361 | 1,04E-01 |
| | | | netrank | netwalk | 0,2541 | -0,0432 | 0,067295 | 0,3445 | 0,0290 | 3,38E-02 |
| | | **BP** | maxlink | netprop | 0,3477 | 0,0031 | 1,92E-02 | 0,3565 | 0,0913 | 3,72E-40 |
| | | | maxlink | netrank | 0,3477 | 0,0693 | 6,88E-13 | 0,3565 | 0,0050 | 6,88E-01 |
| | | | maxlink | netwalk | 0,3477 | 0,0333 | 6,20E-06 | 0,3565 | 0,0580 | 6,03E-16 |
| | | | netprop | netrank | 0,3446 | 0,0662 | 6,92E-10 | 0,2651 | -0,0863 | 7,10E-54 |
| | | | netprop | netwalk | 0,3446 | 0,0302 | 6,18E-03 | 0,2651 | -0,0333 | 2,10E-13 |
| | | | netrank | netwalk | 0,2784 | -0,0361 | 1,85E-04 | 0,3515 | 0,0530 | 8,38E-22 |
| | **101-300** | **CC** | maxlink | netprop | 0,3127 | -0,0277 | 0,531937 | 0,7130 | 0,1557 | 3,41E-09 |
| | | | maxlink | netrank | 0,3127 | 0,0129 | 0,357685 | 0,7130 | 0,0217 | 8,65E-02 |
| | | | maxlink | netwalk | 0,3127 | -0,0159 | 0,731508 | 0,7130 | 0,1005 | 6,92E-06 |
| | | | netprop | netrank | 0,3404 | 0,0406 | 0,031968 | 0,5573 | -0,1340 | 2,63E-04 |
| | | | netprop | netwalk | 0,3404 | 0,0118 | 0,531937 | 0,5573 | -0,0552 | 6,22E-02 |
| | | | netrank | netwalk | 0,2997 | -0,0289 | 0,087026 | 0,6913 | 0,0788 | 1,69E-02 |
| | | **MF** | maxlink | netprop | 0,4251 | 0,0055 | 0,190347 | 0,5245 | 0,1686 | 1,00E-09 |
| | | | maxlink | netrank | 0,4251 | 0,1217 | 0,000262 | 0,5245 | 0,0971 | 5,05E-03 |
| | | | maxlink | netwalk | 0,4251 | 0,0694 | 0,006258 | 0,5245 | 0,1153 | 1,73E-05 |
| | | | netprop | netrank | 0,4196 | 0,1162 | 0,007248 | 0,3559 | -0,0715 | 5,05E-03 |
| | | | netprop | netwalk | 0,4196 | 0,0639 | 0,10625 | 0,3559 | -0,0533 | 6,83E-02 |
| | | | netrank | netwalk | 0,3034 | -0,0523 | 0,056638 | 0,4274 | 0,0182 | 2,98E-01 |
| | | **BP** | maxlink | netprop | 0,3789 | -0,0430 | 1,67E-05 | 0,5057 | 0,1643 | 3,80E-48 |
| | | | maxlink | netrank | 0,3789 | 0,0628 | 3,09E-08 | 0,5057 | -0,0004 | 4,07E-01 |
| | | | maxlink | netwalk | 0,3789 | -0,0009 | 7,40E-01 | 0,5057 | 0,0918 | 2,29E-15 |
| | | | netprop | netrank | 0,4219 | 0,1058 | 5,58E-30 | 0,3414 | -0,1647 | 1,48E-56 |
| | | | netprop | netwalk | 0,4219 | 0,0421 | 8,67E-07 | 0,3414 | -0,0725 | 7,38E-19 |
| | | | netrank | netwalk | 0,3161 | -0,0637 | 1,05E-13 | 0,5061 | 0,0922 | 1,30E-19 |

**Supplemental Figure 1**. **Distribution of GO-terms.** Number of GO-terms for each gene in the subset of GO-terms with 10 to 300 genes (within range 10-300) and for all other genes (outside range 10-300) are illustrated as a histogram.

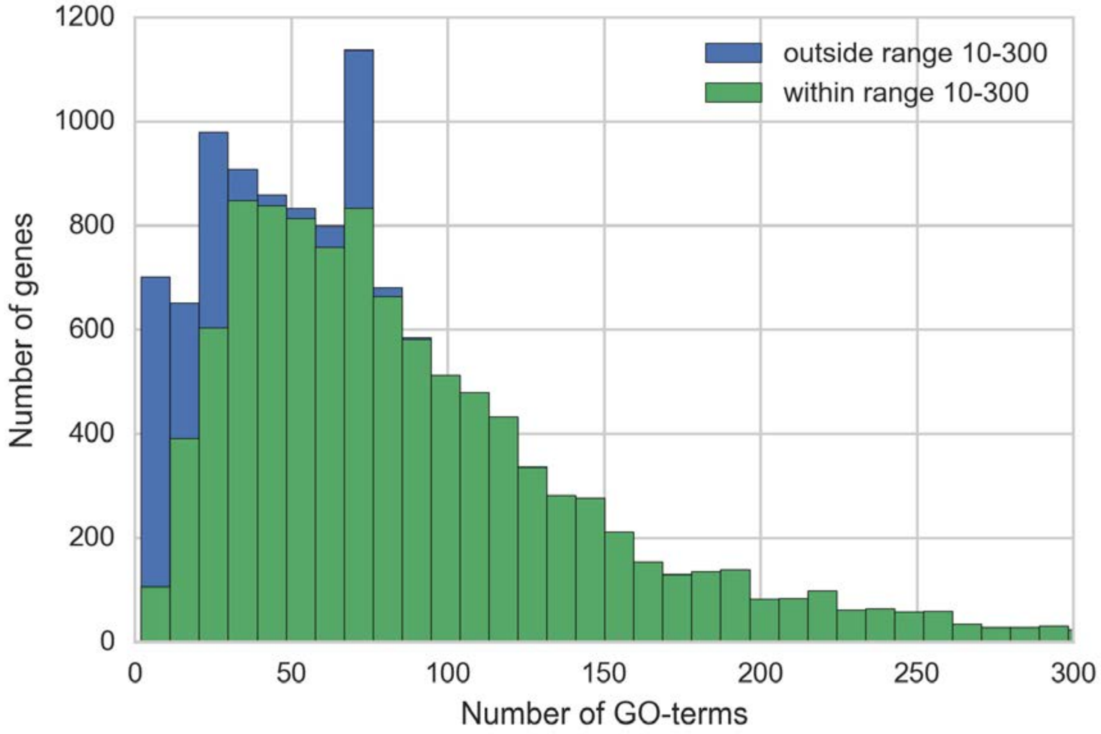

**Supplemental Figure 2**. **Jaccard Normalized Term Overlap vs Shortest Path.** A random sample of 1000 nodes was selected from the FunCoup network. Shortest Path, $d(u, v)$, between pairs of nodes u, v was calculated for all pairwise combinations of nodes in the sample and Jaccard normalized GO-term overlap for the pair $sim_{jnto}(u, v)$ was calculated as follows:

$$sim_{jnto}(u, v) = \frac{|terms_u \cap terms_v|}{|terms_u \cup terms_v|}$$

Sampling was repeated 1000 times and the distribution of $sim_{jnto}(u, v)$ is illustrated as a boxplot for each $d(u, v)$. The box in the boxplot is showing the median and the quartiles (1 and 3) of the dataset while the whiskers extend out to 1.5 the size of the inter-quartile range. The distribution of $sim_{jnto}(u, v)$ for nodes without a path connecting them is illustrated in the "*No path*" category.

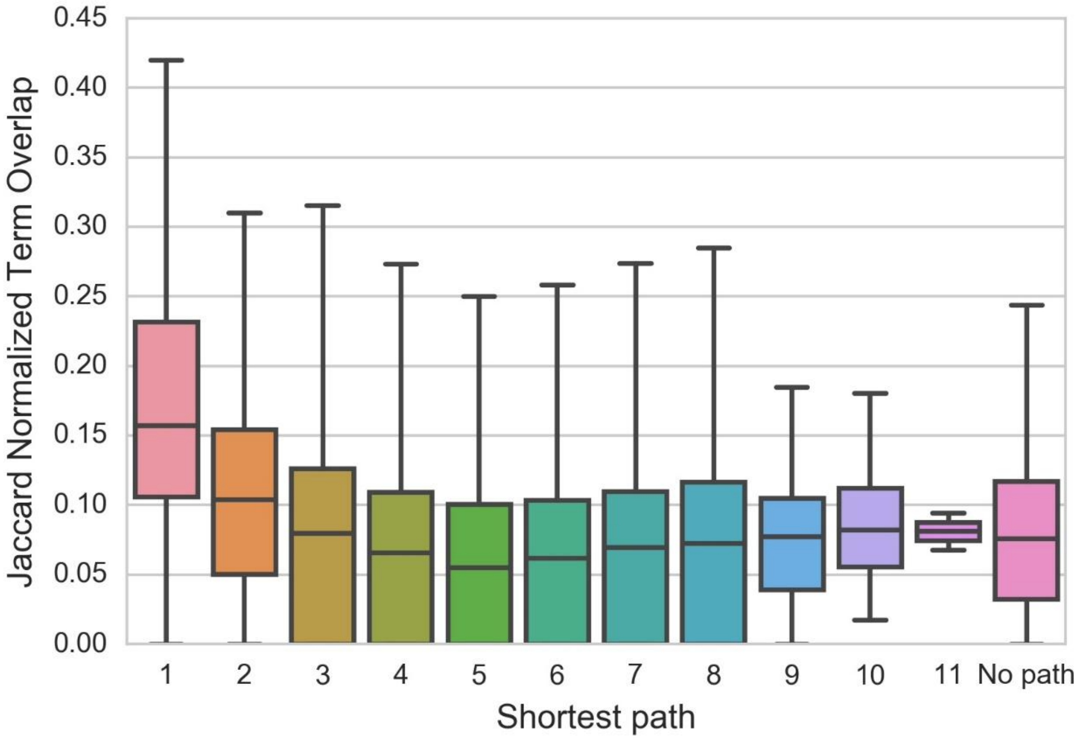

**Supplemental Figure 3. Number of GO-terms per node degree.** All genes in the human genome were divided into bins according to their network node degree (number of connections in FunCoup). The bin '0' corresponds to genes not present in FunCoup. The distribution of number of GO-terms for genes in each bin is illustrated as a boxplot, where the box is showing the median and the quartiles (1 and 3) of the dataset while the whiskers extend out to 1.5 the size of the inter-quartile range.

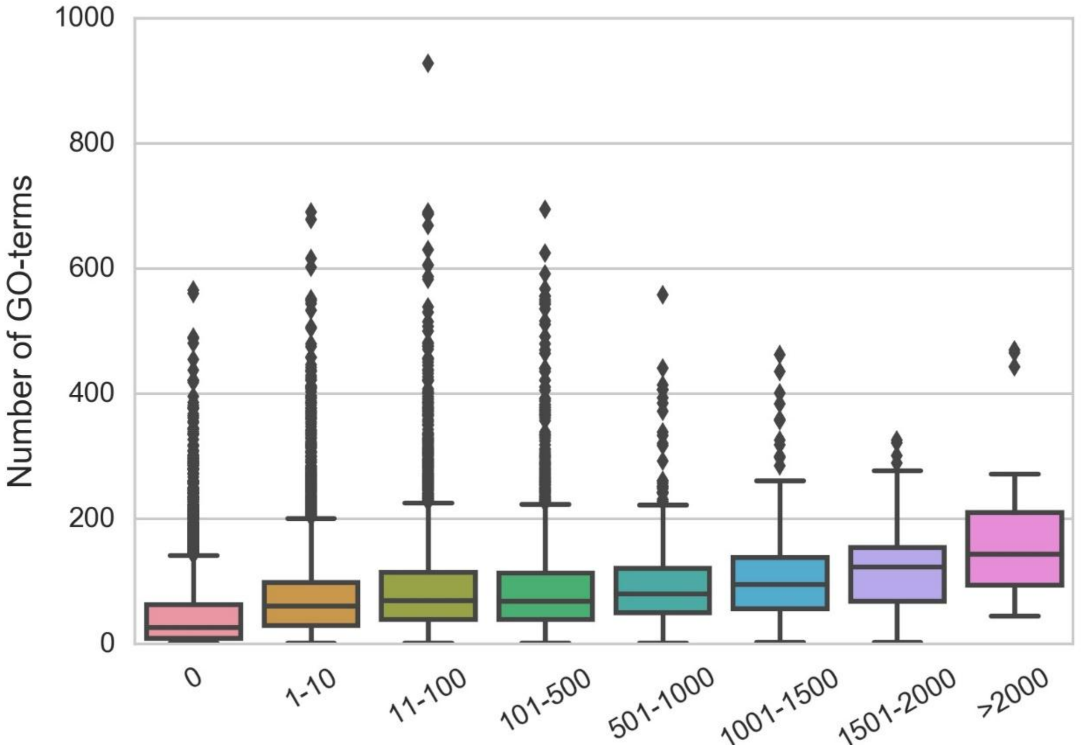

Supplement: Supplementary Information [file srep46598-s1.pdf]
